# Early change in lean tissue mass after peritoneal dialysis: prevalence, risk factors, and clinical implications

**DOI:** 10.1186/s12882-025-04350-6

**Published:** 2025-07-22

**Authors:** Lixing Xu, Jack Kit-Chung Ng, Gordon Chun-Kau Chan, Winston Wing-Shing Fung, Kai-Ming Chow, Cheuk-Chun Szeto

**Affiliations:** 1 Carol & Richard Yu Peritoneal Dialysis Research Centre, Department of Medicine & Therapeutics, The Chinese University of Hong Kong, Shatin, Hong Kong China; 2 Li Ka Shing Institute of Health Sciences (LiHS), Faculty of Medicine, The Chinese University of Hong Kong, Shatin, Hong Kong China; 3 Department of Medicine & Therapeutics, Prince of Wales Hospital, The Chinese University of Hong Kong, Shatin, NT, Hong Kong China

**Keywords:** Renal failure, Malnutrition, Sarcopenia, Frailty

## Abstract

**Background:**

Muscle wasting has been linked to negative clinical outcomes in patients undergoing peritoneal dialysis (PD). However, it remains unclear how early changes in muscle mass after the initiation of PD affects the clinical outcome. This study aimed to identify factors influencing changes in lean tissue mass (LTM) over six months in new PD patients and to assess their prognostic significance.

**Methods:**

We conducted a study of 90 new PD patients. Over a six-month period, we recorded changes in LTM and adipose tissue mass (ATM) using bioimpedance spectrometry. Outcome measures included patient and technique survival rates.

**Results:**

After 6 months of PD, body weight and body mass index remained unchanged, but 42 patients (46.7%) had decrease in LTM for ≥ 1 kg. The percentage of LTM (LTMp) also dropped from 63.4 ± 13.6% to 61.5 ± 13.4% (*p* = 0.006), accompanied by an increase in ATM. Multiple linear regression models showed a strong correlation between changes in LTM and ATM; for every 1 kg increase in ATM, there was a 1.01 kg decrease in LTM (95% confidence interval: 0.797 to 0.855, *p* < 0.0001). However, the change in LTM during the first six months of PD was not associated with patient or technique survival rates.

**Conclusion:**

Although the overall body weight was static, there was a trend of reduction in LTM and a concommitant increase in ATM during the first 6 months of PD. However, these changes were not associated with adverse clinical outcome.

**Supplementary Information:**

The online version contains supplementary material available at 10.1186/s12882-025-04350-6.

## Introduction

Sarcopenia refers to the loss of skeletal muscle mass and strength, which is prevalent among the elderly and individuals with chronic conditions, such as those undergoing chronic dialysis [1]. Numerous studies have demonstrated that the reduction in skeletal muscle is linked to negative consequences like falls, decreased function, frailty, and increased mortality [1]. In patients receiving dialysis, sarcopenia plays a role in their reduced functionality and higher morbidity rates [2, 3]. In a systematic review and meta-analysis, Wathanavasin et al. [4] showed that sarcopenia was significantly associated with higher mortality risk and cardiovascular events dialysis patients.

Lean tissue mass (LTM), which can be readily determined by bioimpedance spectroscopy (BIS), is now commonly used as the measurement of skeletal muscle mass [5]. BIS measures the resistances of body tissues to electrical current at various frequencies, which were then used to estimate the muscle mass and other body compositions and enable the diagnosis of sarcopenia [6, 7]. This method is particularly useful in the CKD population, as it provides a rapid and non-invasive approach for the assessment of muscle mass as well as body fluid status.

LTM has been found to have prognostic relevance in patients with and without kidney disease. In older adults, a low LTM was associated with increased risk of cognitive function imparement and mortality [8]. In patients with stage 4 and 5 CKD, a low LTM index (LTI) was found to be an independent risk factor for mortality [9]. In hemodialysis patients, progressive loss of LTM has been shown to be related to worse quality of life and increased overall mortality [10]. Published data on LTM in peritoneal dialysis (PD) patients, however, are limited. In a study of 160 PD patients, Kim et al. noted that low LTI at baseline predicted all-cause mortality, but the association became insignificant when cardiovascular factors were included in the multivariate analysis [11]. In this study, however, changes in LTI and fat tissue index (FTI) after 2 years of PD were strongly associated with all-cause mortality than single values in LTI and FTI [11].

In clinical practice, it is imperative to assess the prognostic significance of changes in LTM following patient stabilization on PD rather than the change after an extended period of dialysis. Our present study aims to examine the factors influencing LTM changes after a 6-month period of PD and to elucidate the prognostic implications of these changes.

### Patients and methods

The study was approved by the Joint Chinese University Hong Kong-New Territories East Cluster Clinical Research Ethics Committee (approval number CRE-2023.363). All procedures in this study followed the guidelines outlined in the Declaration of Helsinki.

### Patient selection

This study is a retrospective analysis of a prospective observational cohort on incident adult PD patients at a single center. Patients who had failed chronic hemodialysis, with pacemaker or metallic prosthesis, who were scheduled for living donor kidney transplant or transferal to other renal centers within 6 months were excluded. Baseline multi-frequency bioimpedance spectroscopy, dialysis adequacy, and other laboratory tests were performed around 4 weeks after patients were stable on PD and then repeated 6 months later. All patients provided written inform consent for the study.

### Multi-frequency bioimpedance spectroscopy

As described previously [12], we assessed the lean tissue mass (LTM) using the multi-frequency bioimpedance spectroscopy study. Briefly, electrodes were attached to the patient’s right hand and right foot while in a supine position. The Body Composition Monitor (BCM, Fresenius Medical Care, Germany) was used to measure LTM. Percentage of LTM (LTMp) was computed from LTM and body weight. During the bioimpedance study, we also measured the adipose tissue mass (ATM), volume of overhydration (OH), total body water (TBW), extracellular water (ECW), and intracellular water (ICW). Bioimpedance spectroscopy study was performed during PD fluid dwell because peritoneal dialysate had minimal effect on the measurements according to our previous study [13].

### Dialysis adequacy and other laboratory indices

The assessment of dialysis adequacy was performed by 24-hour dialysate and urine collection as previously described [14], and the total weekly Kt/V was calculated accordingly. The residual glomerular filtration rate (GFR) was determined by the average of 24-hour urinary urea and creatinine clearances [15]. Serum albumin levels were measured by the bromocresol purple method [16]. Other laboratory tests, including routine biochemistry, haemoglobin level, serum iron, total iron binding capacity, and ferritin levels, were performed as part of the routine clinical care.

### Follow up and outcome parameters

After the second bioimpedance spectroscopy measurement at 6 months, all patients were further followed till December 2023. During the follow-up period, the clinical management was decided by an individual clinician and not affected by the study. Primary outcome measuress of this study includes patient survival, technique survival, peritonitis-free survival, peritonitis rate, number of hospital admission, and the total duration of hospitalizations. The peritonitis rate was reported as the number of peritonitis episodes per patient per year as previously described [17]. In this study, technique failure was defined as transferral to hemodialysis, receiving kidney transplant, or death, while transferral to other dialysis centers or recovery of renal function were considered as censoring events.

### Statistical analysis

Statistical analysis was performed using the software SPSS (version 28.0. IBM Corporation, Armonk, NY) and GraphPad Prism (version 10.1.1, GraphPad Software, CA). Data are presented as the mean ± standard deviation unless specified otherwise. The Pearson’s correlation coefficient was employed to investigate the association between the correlation between LTM and other nutritional and biochemical parameters. Similarly, the correlation between changes in LTM (∆LTM) and changes in other parameters after 6 months of PD was also analyzed by the Pearson’s correlation coefficient. Multivariate linear regression models were constructed to identify independent factors associated with ∆LTM and ∆LTMp. In the linear regression models, we included sex, age, and variables with p values < 0.1 in the univariate linear regression analysis. The Kaplan-Meier method was utilized to present the data of patient, technical, and peritonitis-free survival rates. Patients were grouped in quartiles according to their ∆LTM, and the survival rates were compared by the log-rank test. The peritonitis rate, number of hospital admission, and duration of hospitalization between quartiles were compared by the Kruskal Wallis test. A p value less than 0.05 was taken as statistically significant. All probabilities were two-tailed.

## Results

We studied 90 incident PD patients. Their baseline demographics and clinical are summarized in Table 1. Multifrequency bioimpedance spectroscopy, as well as other standard biochemical tests, were performed at baseline and then repeated 6 months after PD. The results are summarized in Table 2.

### Change in body mass after 6 months

After PD for 6 months, 42 (46.7%) had decrease in LTM for ≥ 1 kg, 33 (36.7%) had decrease for ≥ 2 kg. On average, there was a significant decrease in lean tissue mass (LTM) from 38.6 ± 9.9 to 37.7 ± 9.3 kg (paired Student’s t test, *p* = 0.041) and lean tissue percentage (LTMp) from 63.4 ± 13.6% to 61.5 ± 13.4% (*p* = 0.006), but a concommitant increase in adipose tissue mass (ATM) from 19.1 ± 9.3 to 20.6 ± 9.4 kg (*p* = 0.002). As a result, there was no significant change in body weight or body mass index, and the volume of overhydration remained similar during this period (Table 2). The baseline clinical and biochemical characteristics for patients with decrease in LTM ≥ 1 kg and < 1 kg are compared in Supplementary Table 1. The internal correlation between body weight and bioimpedance spectroscopy parameters are summarized in Table 3. In essence, baseline LTM and LTMp were inversely correlated with ATM and E/I. Similarly, after PD for 6 months, the change in LTM and LTMp had close inverse correlations with the concommitant changes in ATM and E/I ratio. Notably, the change in body weight was significantly correlated with the change in ATM but inversely correlated with the change in LTMp.

Multiple linear regression model was further constructed to identify factors associated with changes in LTM after 6 months of PD. In essence, the change in body weight, ATM, and E/I ratio were the only independent factors associated with the change in LTM (Table 4; full model in Supplementary Table 2). In this model, a reduction in LTM by 1 kg was associated with a corresponding increase in ATM by 1.01 ± 0.42 kg, indicating a reciprocal change of the two parameters. Similar findings were observed when multiple linear regression model was constructed for the change in LTMp, with the change in body weight, ATM, and E/I ratio being the only independent predictors (Supplementary Table 3), and a reciprocal finding was observed when multiple linear regression model was constructed for the change in ATM, with the change in body weight, LTM, and overhydration volume being the only independent predictors (Supplementary Table 4).

### Patient and technical survival

**Fig. 1:**
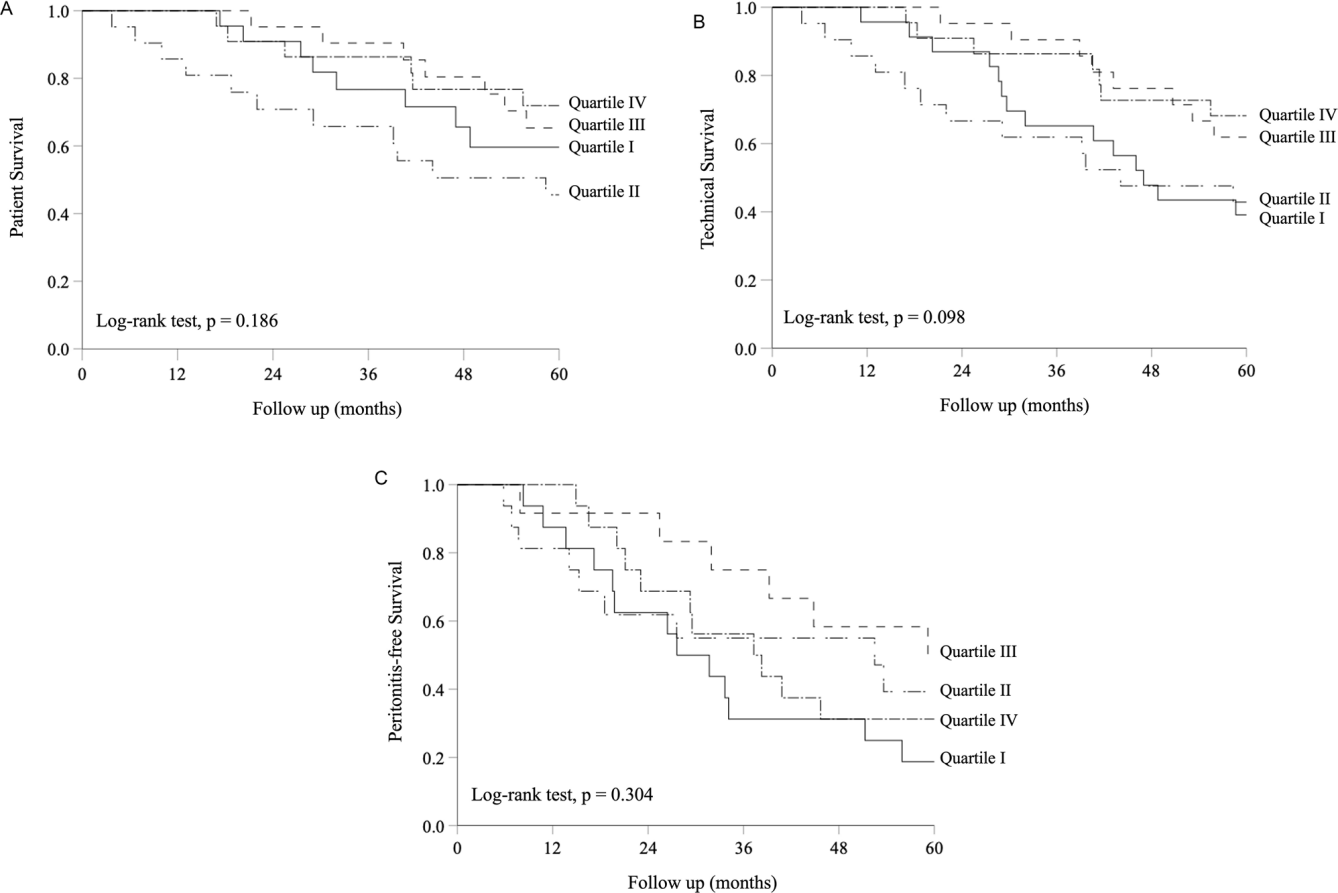
Kaplan-Meier plot of (**A**) patient survival; (**B**) technique survival; and (**C**) peritonitis-free survival. Patients were divided into quartiles according to the change in lean tissue mass (LTM) over the first 6 months of peritoneal dialysis, with quartile I the lowest values (i.e. most reduction in LTM). Data were compared by the log-rank test

During a median follow-up of 67.5 (IQR 37.9 to 99.9) months, 65 (72.2%) patients died. The causes of death were infections other than peritonitis (17 cases), ischemic heart disease (15 cases), cerebral vascular accident (7 cases), peritonitis (14 cases), sudden cardiac death (7 cases), termination of dialysis (1 case), and other specific causes (4 cases). During that period, 12 patients were converted to hemodialysis, 6 patients had kidney transplantation, and 3 patients were transferred to other centres. The relation between the change in LTM after 6 months of PD, divided into quartiles, and patient and technique survival rates are summarized in Kaplan Meier plots (Fig. 1A). The 2-year patient survival rates for quartiles I to IV of the change in LTM were 90.9%, 70.8%, 95.2%, and 86.3%, respectively (log-rank test *p* = 0.186). The 2-year technical survival rates for quartiles I to IV of the change in LTM were 87.0%, 66.7%, 95.2%, and 86.4%, respectively (log-rank text *p* = 0.098) (Fig. 1B). The result of survival analysis remained similar when follow up was censored at 24 months.

### Peritonitis and hospitalization

During the study period, there were 181 peritonitis episodes recorded in 62 patients; 28 patients (31.1%) were peritonitis-free. The overall peritonitis rate was 0.40 episode per patient-year follow up. The 2-year peritonitis-free survival rate for quartiles I to IV of the change in LTM were 56.2%, 61.6%, 83.3%, and 68.8%, respectively (log-rank test *p* = 0.304) (Fig. 1C). The peritonitis rate for quartiles I to IV of the change in LTM were 0.31, 0.51, 0.18, and 0.49 episodes per patient-year, respectively (*p* = 0.06). There was no significant association between the peritonitis rate and the change in LTM, LTMp, or ATM (Supplementary Table 5).

During the study period, there were 650 hospital admissions, with a total of 4554 days of hospital stay. The hospitalization rates were 1.50, 1.00, 0.99, and 1.53 per patient-year for quartiles I to IV of the change in LTM, respectively (*p* = 0.103). The durations of hospital stay were 13.2, 7.1, 6.5, and 11.8 days per patient-year for quartiles I to IV of the change in LTM, respectively. There was no significant association between the number of hospital admission or duration of hospitalization with the change in LTM, LTMp, or ATM (Supplementary Table 5).

## Discussion

In this present study, we reviewed the changes in body composition and nutritional indicators during the first 6 months of PD. Unlike previous studies that often cover extended periods, our focus was on the early period when the patients were stabilized on PD. Our findings confirmed that muscle wasting and fat gain were common and occurred simultaneously in the early period after PD was initiated, which is consistent with the previous report of Guida et al. [18]. We found that in addition to weight loss, increase in adipose tissue mass and rise in extra-to-intracellular water ratio were independent predictors of lean tissue mass loss. In contrast, no baseline demographic or biochemical parameters predict muscle wasting in the early months after PD.

The association between the changes in ATM and LTM is expected as similar findings have been reported in hemodialysis patients [19]. Based on our data, every 1 kg increase of ATM is asociated with 1.01 kg decrease in LTM (see Table 4). As a result, the overall body weight remained static. We also observed a significant inverse correlation between the change in E/I ratio and LTM. This is an expected finding because LTM measured by BIS is derived from the volume of intracellular water water (ICW) [20], which is the denominator of the E/I ratio. Although the E/I ratio is an indicator of fluid overload [21], we found no association between the change in LTM and overhydration volume (OH), suggesting that the correlation between the changes in E/I ratio and LTM is mathematical coupling rather than a biological phenomenon.

In this study, we did not find any baseline biochemical parameter or their serial chang related to the number of peritonitis episodes, number of hospitalizations, or the duration of hospitalization. Similarly, we did not find any association between reduction in muscle mass during the first 6 months of PD and the subsequent patient, technical, or peritonitis-free survival. On the first glance, our finding appears to be different from the previous studies [11, 23]. Notably, Kim et al. [11] reported that muscle wasting was associated with an increase in patient mortality, while Davenport [23] showed that weight change after PD was associated with the subsequent risk of peritonitis. Nonetheless, there are important differences between our studies from previous ones. Notably, both of the previous studies examined the changes of muscle mass over 2 years [11, 23], while we focused on the change in the first 6-month of PD. In addition, baseline LTM is negatively associated with its subsequent change in 6 months by univariate analysis. It was possible that patients who experienced greater LTM reduction were more likely to have a higher LTM at baseline, which negated the effect on patient mortality [22]. Since both our study and the previously ones had relatively small sample sizes, further studies are required to confirm the findings.

The present study has several limitations. First, the sample size was modest and outliers were not uncommon in our data set. Based on our observation, it is estimated that a sample size of 277 would be required to have 80% power for confirming a significant effect [24]. Since we only examined the change in LTM over the first 6 months of PD – and previous studies only examined the change in two time points [11, 23] – serial changes in muscle mass over extended period of time would be an important area to explore. Although we found an inverse correlation between muscle mass and fat mass, we did not classify the adipose tissue to subcutaneous and visceral fat, making it unclear which type of adipose tissue is actually replacing the muscle. More importantly, other factors that may affect muscle and adipose tissue mass, notably, physical activity, have not been assessed.

In conclusion, we found a trend of reduction in LTM and a concommitant increase in ATM during the first 6 months of PD. However, the changes during the first 6 months of PD was not associated with an adverse clinical outcome subsequently. Since the overall body weight remained static, our result highlights the need of comprehensive body composition examination in PD patients rather than relying on the simple BMI.

**Table 1:** Baseline demographic and clinical characteristics

No. of patients	90
Sex (male: female)	44:46
Age (years)	60.8 ± 10.8
Height (cm)	161.5 ± 8.7
Blood pressure (mmHg)	
Systolic	134.1 ± 21.6
Diastolic	72.3 ± 12.7
Renal diagnosis, no. of case (%)	
Diabetic nephropathy	37 (41.1%)
Glomerulonephritis	23 (25.6%)
Hypertension	11 (12.2%)
Urological problem	5 (5.6%)
Polycystic kidney disease	3 (3.3%)
Others or unknown	11 (12.2%)
Major comorbidities, no. of case (%)	
Diabetes mellitus	45 (50.0%)
Coronary artery disease	24 (26.7%)
Cerebrovascular accident	21 (23.3%)
Peripheral vascular disease	6 (6.7%)
Charlson’s comorbidity index	6.1 ± 2.4
Type of PD, no. of case (%)	
Machine-assisted automated PD	15 (16.7%)
Low GDP solution	21 (23.3%)

PD, peritoneal dialysis; GDP, glucose degradation products

**Table 2:** Baseline bioimpedance and biochemical information

	0 month	6 months	*P* value
Body weight (kg)	61.1 ± 11.3	61.9 ± 11.2	0.077
Body mass index (kg/m^2^)	23.4 ± 3.8	23.7 ± 3.6	0.248
Bioimpedance parameters			
Lean tissue mass (kg)	38.6 ± 9.9	37.7 ± 9.3	0.041
Lean tissue percentage (%)	63.4 ± 13.6	61.5 ± 13.4	0.006
Adipose tissue mass (kg)	19.1 ± 9.3	20.6 ± 9.4	0.002
Overhydration (L)	3.3 ± 3.3	3.1 ± 3.0	0.286
E/I ratio	0.97 ± 0.18	0.97 ± 0.18	0.912
Hemoglobin (g/dL)	9.4 ± 1.5	9.6 ± 1.6	0.262
Serum albumin (g/L)	35.3 ± 4.8	35.3 ± 5.0	0.873
Fasting plasma glucose (mmol/l)	6.1 ± 2.1	6.6 ± 2.0	0.226
Lipid profile (mmol/l)			
Total cholesterol	5.2 ± 1.6	5.2 ± 1.5	0.467
LDL cholesterol	3.1 ± 1.3	3.1 ± 1.2	0.827
HDL cholesterol	1.3 ± 0.4	1.3 ± 0.5	0.211
Triglyceride	1.8 ± 1.2	2.0 ± 1.9	0.433
Total weekly Kt/V	2.2 ± 0.6	2.1 ± 0.6	0.188
Residual GFR (ml/min/1.73m^2^)	3.9 ± 2.8	2.8 ± 2.5	0.008
Iron profile			
Plasma iron (µmol/l)	12.6 ± 5.8	13.1 ± 5.4	0.845
Plasma TIBC (µmol/l)	37.4 ± 7.4	37.7 ± 8.1	0.585
Iron saturation (%)	0.3 ± 0.2	0.4 ± 0.1	0.979
Serum ferritin (ng/mL)	1124.5 ± 1004.7	1122.5 ± 1125.0	< 0.0001

E/I ratio, extracellular to intracellular volume ration; LDL, low density lipoprotein; HDL, high density lipoprotein; Kt/V, dialysis adequacy; GFR, glomerular filtration rate; TIBC, total iron binding capacity

**Table 3:** Internal correlation matrices between body composition parameters **baseline body composition**

	Body weight	LTM	LTMp	ATM	OH
**(A) Baseline body composition**					
LTM	*r* = 0.537, *p* < 0.0001				
LTMp	*r* = 0.187, *p* = 0.083	*r* = 0.720, *p* < 0.0001			
ATM	*r* = 0.396, *p* < 0.0001	*r* = -0.494, *p* < 0.0001	*r* = − 0.905, *p* < 0.0001		
OH	*r* = 0.482, *p* < 0.0001	*r* = 0.248, *p* = 0.021	*r* = 0.121, *p* = 0.265	*r* = -0.068, *p* = 0.532	
E/I ratio	*r* = 0.371, *p* < 0.0001	*r* = -0.247, *p* = 0.021	*r* = -0.566, *p* < 0.0001	*r* = 0.316, *p* = 0.003	*r* = 0.833, *p* < 0.0001
**(B) Change in body composition in 6 months**					
LTM	*r* = 0.193, *p* = 0.073				
LTMp	*r* = -0.319, *p* = 0.003	*r* = 0.851, *p* < 0.0001			
ATM	*r* = 0.344, *p* = 0.001	*r* = -0.769, *p* < 0.0001	*r* = -0.890, *p* < 0.0001		
OH	*r* = 0.354, *p* = 0.001	*r* = 0.142, *p* = 0.188	*r* = -0.079, *p* = 0.467	*r* = -0.291, *p* = 0.007	
E/I ratio	*r* = 0.423, *p* < 0.0001	*r* = -0.226, *p* = 0.036	*r* = -0.448, *p* < 0.0001	*r* = 0.093, *p* = 0.396	*r* = 0.895, *p* < 0.0001

**Table 4:** Summary of multi-variable linear regression models on the factors associated with the change in lean tissue mass

	Beta	95% CI	*P* values
Sex	0.004	-0.380 to 0.461	0.846
Age	0.017	-0.008 to 0.024	0.330
Baseline parameters			
Lean tissue mass	0.013	-0.019 to 0.035	0.571
Adipose tissue mass	0.006	-0.014 to 0.012	0.680
Change in 6 months			
Weight	0.803	0.850 to 0.936	< 0.0001
Adipose tissue mass	-1.011	-0.855 to – 0.797	< 0.0001
E/I ratio	-0.425	-16.092 to -13.965	< 0.0001
FPG	0.001	-0.054 to 0.058	0.941

CI, confidence interval; E/I ratio, extracellular to intracellular fluid volume ration; FPG, fasting plasma glucose.

## Electronic supplementary material

Below is the link to the electronic supplementary material.

Supplementary Material 1

## Data Availability

The original data supporting the findings of this study are openly available in Github repository at: https://github.com/szetocc/serial_LTM.
