# Supplementary material for: Early change in lean tissue mass after peritoneal dialysis: prevalence, risk factors, and clinical implications
Source: BMC Nephrol. 2025 Jul 22;26:411. doi: 10.1186/s12882-025-04350-6 (PMC12281812; doi:10.1186/s12882-025-04350-6)
Supplement: Supplementary file 1 — Supplementary Material 1 [file 12882_2025_4350_MOESM1_ESM.docx]

# Supplementary Table 1. Comparison of clinical and biochemical parameters between patients with LTM reduction over 1kg and the others

|  | decrease in LTM over 6 months | |  |
| --- | --- | --- | --- |
|  | ≥1 kg | < 1 kg | P value |
| No. of patients | 42 | 48 |  |
| Sex (male: female) | 19 : 23 | 25 : 23 | 0.473* |
| Age (years) | 62.3 ± 10.3 | 59.5 ± 11.0 | 0.284 |
| Renal diagnosis, no. of case (%) |  |  | 0.632* |
| diabetic nephropathy | 18 (42.9%) | 19 (39.6%) |  |
| glomerulonephritis | 8 (19.0%) | 15 (31.3%) |  |
| hypertension | 5 (11.9%) | 6 (12.5%) |  |
| urological problem | 4 (9.5%) | 1 (2.1%) |  |
| polycystic kidney disease | 2 (4.8%) | 1 (2.1%) |  |
| others or unknown | 5 (11.9 %) | 6 (12.5%) |  |
| Major comorbidities, no. of case (%) |  |  |  |
| diabetes mellitus | 22 (52.4%) | 23 (47.9%) | 0.673* |
| coronary artery disease | 13 (31.0%) | 11 (22.9%) | 0.390* |
| cerebrovascular accident | 8 (19.0%) | 13 (27.1%) | 0.369* |
| peripheral vascular disease | 3 (7.1%) | 3 (6.3%) | 0.865* |
| Charlson’s comorbidity index | 6.2 ± 2.6 | 6.1 ± 2.2 | 0.995 |
| Height (cm) | 161.6 ± 9.2 | 161.4 ± 8.1 | 0.999 |
| Systolic Blood Pressure (mmHg) | 133.0 ± 24.2 | 134.1 ± 19.4 | 0.621 |
| Diastolic Blood Pressure (mmHg) | 70.9 ±13.0 | 72.9 ± 12.5 | 0.358 |
| Body weight (kg) | 62.3 ± 11.0 | 60.1 ± 11.3 | 0.383 |
| Body mass index (kg/m^2^) | 23.9 ± 3.9 | 23.0 ± 3.6 | 0.259 |
| Bioimpedance parameters |  |  |  |
| lean tissue mass (kg) | 41.5 ± 10.8 | 35.9 ± 7.9 | 0.009 |
| lean tissue percentage (%) | 66.9 ± 14.2 | 60.1 ± 12.0 | 0.021 |
| adipose tissue mass (kg) | 17.3 ± 9.3 | 20.7 ± 8.8 | 0.101 |
| overhydration (L) | 3.3 ± 3.5 | 3.3 ± 3.0 | 0.959 |
| E/I ratio | 1.0 ± 0.2 | 1.0 ± 0.2 | 0.448 |
| Hemoglobin (g/dL) | 9.3 ± 1.6 | 9.6 ± 1.4 | 0.411 |
| Serum albumin (g/L) | 35.1 ± 4.5 | 35.4 ± 4.9 | 0.764 |
| Fasting plasma glucose (mmol/l) | 5.8 ± 2.4 | 6.3 ± 1.8 | 0.382 |
| Lipid profile (mmol/l) |  |  |  |
| total cholesterol | 5.2 ± 1.5 | 5.2 ± 1.6 | 0.860 |
| LDL cholesterol | 3.0 ± 1.2 | 3.1 ± 1.3 | 0.808 |
| HDL cholesterol | 1.3 ± 0.5 | 1.3 ± 0.4 | 0.637 |
| triglyceride | 1.8 ± 1.3 | 1.8 ± 1.1 | 0.929 |
| Total weekly Kt/V | 2.2 ± 0.6 | 2.2 ± 0.7 | 0.854 |
| Residual GFR (ml/min/1.73m^2^) | 4.2 ± 2.8 | 3.5 ± 2.7 | 0.426 |
| Iron profile |  |  |  |
| plasma iron (μmol/l) | 12.6 ± 5.6 | 12.7 ± 5.8 | 0.905 |
| plasma TIBC (μmol/l) | 36.9 ± 6.5 | 38.0 ± 8.0 | 0.557 |
| iron saturation (%) | 34.8 ± 18.7 | 34.9 ± 18.7 | 0.999 |
| serum ferritin (ng/mL) | 1191.2 ± 911.9 | 1299.6 ± 1070.2 | 0.690 |

LTM, lean tissue mass; E/I ratio, extracellular to intracellular volume ratio; LDL, low density lipoprotein; HDL, high density lipoprotein; Kt/V, dialysis adequacy; GFR, glomerular filtration rate; TIBC, total iron binding capacity. Data were compared by unpaired Student’s t test or *Chi square test.

# Supplementary Table 2. Full Linear regression models on the factors associated with the change in lean tissue mass

|  | Uni-variable | | Multi-variable | | |
| --- | --- | --- | --- | --- | --- |
|  | Beta | P values | Beta | 95% CI | P values |
| Sex | 0.010 | 0.929 | 0.004 | -0.380 to 0.461 | 0.846 |
| Age | -0.130 | 0.229 | 0.017 | -0.008 to 0.024 | 0.330 |
| Height | -0.047 | 0.663 |  |  |  |
| Baseline parameters |  |  |  |  |  |
| Weight | -0.149 | 0.170 |  |  |  |
| Systolic BP | -0.030 | 0.781 |  |  |  |
| Diastolic BP | 0.086 | 0.429 |  |  |  |
| Charlson’s score | -0.135 | 0.212 |  |  |  |
| Lean tissue mass | -0.344 | 0.0001 | 0.013 | -0.019 to 0.035 | 0.571 |
| Adipose tissue mass | 0.213 | 0.049 | 0.006 | -0.014 to 0.012 | 0.680 |
| Overhydration | -0.072 | 0.509 |  |  |  |
| E/I ratio | 0.074 | 0.496 |  |  |  |
| Hemoglobin | 0.022 | 0.846 |  |  |  |
| Albumin | 0.012 | 0.910 |  |  |  |
| FPG | -0.052 | 0.688 |  |  |  |
| Total Cholesterol | 0.009 | 0.944 |  |  |  |
| LDL Cholesterol | 0.001 | 0.996 |  |  |  |
| HDL Cholesterol | -0.035 | 0.792 |  |  |  |
| Triglyceride | 0.044 | 0.739 |  |  |  |
| Kt/V | 0.082 | 0.532 |  |  |  |
| Residual GFR | 0.054 | 0.698 |  |  |  |
| Plasma Iron | -0.061 | 0.651 |  |  |  |
| Plasma TIBC | 0.174 | 0.192 |  |  |  |
| Iron saturation | -0.101 | 0.430 |  |  |  |
| Plasma Ferritin | -0.055 | 0.692 |  |  |  |
| Change in 6 months |  |  |  |  |  |
| Weight | 0.193 | 0.073 | 0.803 | 0.850 to 0.936 | < 0.0001 |
| Systolic BP | -0.043 | 0.692 |  |  |  |
| Diastolic BP | -0.050 | 0.647 |  |  |  |
| Adipose tissue mass | -0.769 | < 0.0001 | -1.011 | -0.855 to – 0.797 | < 0.0001 |
| Overhydration | 0.142 | 0.188 |  |  |  |
| E/I ratio | -0.226 | 0.035 | -0.425 | -16.092 to -13.965 | < 0.0001 |
| Hemoglobin | -0.130 | 0.243 |  |  |  |
| Albumin | -0.122 | 0.269 |  |  |  |
| FPG | -0.322 | 0.036 | 0.001 | -0.054 to 0.058 | 0.941 |
| Total Cholesterol | -0.016 | 0.924 |  |  |  |
| LDL Cholesterol | 0.061 | 0.707 |  |  |  |
| HDL Cholesterol | 0.046 | 0.776 |  |  |  |
| Triglyceride | -0.168 | 0.300 |  |  |  |
| Total weekly Kt/V | -0.067 | 0.711 |  |  |  |
| Residual GFR | -0.133 | 0.501 |  |  |  |
| Plasma Iron | -0.007 | 0.959 |  |  |  |
| Plasma TIBC | -0.007 | 0.963 |  |  |  |
| Iron saturation | 0.033 | 0.959 |  |  |  |
| Serum Ferritin | 0.155 | 0.451 |  |  |  |

CI, confidence interval; BP, blood pressure; E/I ratio, extracellular to intracellular fluid volume ration; FPG, fasting plasma glucose; LDL, low density lipoprotein; HDL, high density lipoprotein; GFR, glomerular filtration rate; TIBC, total iron binding capacity.

# Supplementary Table 3. Linear regression models on the factors associated with the change in lean tissue percentage

|  | Uni variable | | Multi variable | | |
| --- | --- | --- | --- | --- | --- |
|  | Beta | P values | Beta | 95% CI | P values |
| Sex | -0.126 | 0.244 | -0.002 | -1.303 to 1.243 | 0.962 |
| Age | -0.137 | 0.207 | 0.033 | -0.032 to 0.088 | 0.347 |
| Height | -0.128 | 0.236 |  |  |  |
| Baseline Parameters |  |  |  |  |  |
| Weight | -0.054 | 0.620 |  |  |  |
| Systolic BP | 0.113 | 0.296 |  |  |  |
| Diastolic BP | 0.152 | 0.161 |  |  |  |
| Charlson’s Score | -0.060 | 0.583 |  |  |  |
| Lean tissue percentage | -0.370 | 0.0004 | 0.013 | -0.054 to 0.072 | 0.775 |
| Overhydration | 0.033 | 0.792 |  |  |  |
| E/I | 0.193 | 0.074 | 0.042 | -4.355 to 2.117 | 0.327 |
| Hemoglobin | 0.026 | 0.816 |  |  |  |
| Albumin | 0.023 | 0.832 |  |  |  |
| FPG | -0.077 | 0.553 |  |  |  |
| Total Cholesterol | 0.123 | 0.354 |  |  |  |
| LDL Cholesterol | 0.110 | 0.408 |  |  |  |
| HDL Cholesterol | 0.140 | 0.291 |  |  |  |
| Triglyceride | 0.208 | 0.113 |  |  |  |
| Weekly Kt/V | 0.035 | 0.791 |  |  |  |
| Residual GFR | -0.017 | 0.901 |  |  |  |
| Plasma iron | -0.199 | 0.134 |  |  |  |
| Plasma TIBC | 0.172 | 0.198 |  |  |  |
| Iron Saturation | -0.221 | 0.095 | -0.023 | -22.096 to -1.102 | 0.487 |
| Plasma Ferritin | -0.162 | 0.236 |  |  |  |
| Changes in 6 months |  |  |  |  |  |
| Weight | -0.319 | 0.003 | 0.201 | 0.236 to 0.595 | < 0.0001 |
| Systolic BP | -0.109 | 0.315 |  |  |  |
| Diastolic BP | -0.073 | 0.502 |  |  |  |
| Adipose tissue mass | -0.890 | < 0.0001 | -0.936 | -1.388 to -1.158 | < 0.0001 |
| Overhydration | -0.079 | 0.467 |  |  |  |
| E/I | -0.448 | < 0.0001 | -0.393 | -30.413 to -19.794 | < 0.0001 |
| Hemoglobin | -0.092 | 0.410 |  |  |  |
| albumin | -0.021 | 0.852 |  |  |  |
| FPG | -0.233 | 0.148 |  |  |  |
| Total Cholesterol | -0.186 | 0.249 |  |  |  |
| LDL Cholesterol | -0.131 | 0.421 |  |  |  |
| HDL Cholesterol | 0.004 | 0.981 |  |  |  |
| Triglyceride | -0.102 | 0.529 |  |  |  |
| Weekly Kt/V | 0.056 | 0.755 |  |  |  |
| Residual GFR | -0.124 | 0.529 |  |  |  |
| Plasma Iron | 0.062 | 0.670 |  |  |  |
| Plasma TIBC | -0.071 | 0.626 |  |  |  |
| Iron saturation | 0.110 | 0.448 |  |  |  |
| Serum Ferritin | 0.331 | 0.038 | 0.046 | 0.0001 to 0.002 | 0.142 |

CI, confidence interval; BP, blood pressure; E/I ratio, extracellular to intracellular fluid volume ration; FPG, fasting plasma glucose; LDL, low density lipoprotein; HDL, high density lipoprotein; GFR, glomerular filtration rate; TIBC, total iron binding capacity.

Note: baseline ATM is excluded in this model due to its collinearity problem with baseline LTMp.

# Supplementary Table 4. Linear regression models on the factors associated with the change in adipose tissue mass

|  | Uni variable | | Multi variable | | |
| --- | --- | --- | --- | --- | --- |
| Pearson’s | Beta | p values | Beta | 95% CI | P values |
| Sex | -0.124 | 0.256 | -0.003 | -0.127 to 0.061 | 0.475 |
| Age | 0.118 | 0.278 | 0.005 | 0.001 to 0.007 | 0.085 |
| Height | 0.123 | 0.259 |  |  |  |
| Baseline Parameters |  |  |  |  |  |
| Weight | 0.105 | 0.338 |  |  |  |
| Systolic BP | -0.074 | 0.497 |  |  |  |
| Diastolic BP | -0.115 | 0.290 |  |  |  |
| Charlson’s Score | 0.014 | 0.895 |  |  |  |
| Lean tissue mass | 0.379 | < 0.0001 | 0.005 | -0.001 to 0.009 | 0.223 |
| Adipose tissue mass | -0.352 | 0.001 | 0.0001 | -0.001 to 0.001 | 0.999 |
| OH_L | 0.180 | 0.098 | 0.002 | 0.008 to 0.015 | 0.548 |
| E/I | -0.010 | 0.925 |  |  |  |
| Hb | -0.034 | 0.759 |  |  |  |
| albumin | -0.141 | 0.201 |  |  |  |
| FPGlu | 0.075 | 0.571 |  |  |  |
| TChol | -0.033 | 0.804 |  |  |  |
| LDL | -0.015 | 0.909 |  |  |  |
| HDL | 0.128 | 0.337 |  |  |  |
| Triglyceride | -0.154 | 0.248 |  |  |  |
| Kt/V | 0.025 | 0.852 |  |  |  |
| GFR | 0.015 | 0.913 |  |  |  |
| Plasma iron | 0.252 | 0.059 |  |  |  |
| TIBC | -0.150 | 0.266 |  |  |  |
| Iron_sat | 0.265 | 0.047 | -0.005 | -0.375 to 0.026 | 0.086 |
| Ferritin | 0.194 | 0.159 |  |  |  |
| Changes in 6 months |  |  |  |  |  |
| Weight | 0.344 | 0.001 | 0.658 | 0.989 to 1.010 | < 0.0001 |
| Systolic BP | 0.107 | 0.325 |  |  |  |
| Diastolic BP | 0.105 | 0.337 |  |  |  |
| Lean tissue mass | -0.769 | < 0.0001 | -0.780 | -1.015 to -0.997 | < 0.0001 |
| Overhydration | -0.291 | 0.007 | -0.374 | -1.068 to -1.024 | < 0.0001 |
| E/I | 0.093 | 0.396 |  |  |  |
| Hemoglobin | 0.078 | 0.490 |  |  |  |
| albumin | 0.235 | 0.032 | 0.005 | -0.003 to 0.016 | 0.160 |
| FPG | 0.222 | 0.174 |  |  |  |
| Total Cholesterol | 0.061 | 0.713 |  |  |  |
| LDL Cholesterol | 0.063 | 0.702 |  |  |  |
| HDL Cholesterol | 0.019 | 0.909 |  |  |  |
| Triglyceride | -0.017 | 0.919 |  |  |  |
| weekly Kt/V | -0.136 | 0.459 |  |  |  |
| Residual GFR | 0.089 | 0.660 |  |  |  |
| Plasma Iron | -0.105 | 0.473 |  |  |  |
| Plasma TIBC | 0.126 | 0.388 |  |  |  |
| Iron saturation | -0.170 | 0.243 |  |  |  |
| Plasma Ferritin | -0.331 | 0.028 | 0.004 | -0.0001 to 0.0001 | 0.188 |

CI, confidence interval; BP, blood pressure; E/I ratio, extracellular to intracellular fluid volume ration; FPG, fasting plasma glucose; LDL, low density lipoprotein; HDL, high density lipoprotein; GFR, glomerular filtration rate; TIBC, total iron binding capacity.

# Supplementary Table 5. Correlation of peritonitis rate and hospitalization with clinical parameters

|  | Number of peritonitis episodes per year | | Number of hospital admissions per year | | Duration of hospitalization per year | |
| --- | --- | --- | --- | --- | --- | --- |
|  | Pearson’s r | P values | Pearson’s r | P values | Pearson’s r | P values |
| Sex | 0.075 | 0.484 | 0.017 | 0.866 | 0.054 | 0.598 |
| Age | 0.042 | 0.691 | -0.145 | 0.156 | -0.064 | 0.535 |
| Weight | -0.016 | 0.883 | 0.024 | 0.819 | 0.014 | 0.894 |
| Systolic BP | -0.04 | 0.711 | 0.256 | 0.015 | 0.088 | 0.407 |
| Diastolic BP | 0.099 | 0.358 | 0.211 | 0.048 | 0.178 | 0.096 |
| LTM | 0.036 | 0.738 | -0.138 | 0.203 | -0.206 | 0.055 |
| LTMp | 0.035 | 0.745 | -0.127 | 0.243 | -0.201 | 0.062 |
| ATM | 0.031 | 0.777 | 0.170 | 0.118 | 0.155 | 0.154 |
| OH | -0.084 | 0.431 | -0.094 | 0.377 | -0.056 | 0.598 |
| E/I | -0.102 | 0.341 | 0.034 | 0.753 | 0.165 | 0.123 |
| Hemoglobin | -0.147 | 0.179 | -0.129 | 0.238 | -0.104 | 0.342 |
| albumin | -0.118 | 0.278 | -0.071 | 0.793 | -0.138 | 0.202 |
| FPG | 0.215 | 0.182 | -0.270 | 0.092 | -0.082 | 0.615 |
| Total Cholesterol | -0.083 | 0.612 | -0.264 | 0.099 | -0.069 | 0.671 |
| LDL Cholesterol | -0.115 | 0.480 | -0.118 | 0.468 | 0.095 | 0.560 |
| HDL Cholesterol | -0.168 | 0.299 | -0.196 | 0.226 | -0.273 | 0.089 |
| Triglyceride | 0.121 | 0.458 | -0.207 | 0.200 | -0.020 | 0.312 |
| Weekly Kt/V | 0.071 | 0.703 | -0.169 | 0.363 | -0.164 | 0.470 |
| Residual GFR | 0.158 | 0.430 | -0.118 | 0.556 | -0.166 | 0.407 |
| Plasma Iron | -0086 | 0.555 | 0.010 | 0.954 | -0.049 | 0.741 |
| Plasma TIBC | 0.119 | 0.415 | 0.187 | 0.199 | 0.115 | 0.433 |
| Iron saturation | -0.145 | 0.322 | -0.038 | 0.794 | -0.051 | 0.726 |
| Plasma Ferritin | -0.002 | 0.987 | -0.024 | 0.876 | -0.064 | 0.680 |

BP, blood pressure; E/I ratio, extracellular to intracellular fluid volume ration; FPG, fasting plasma glucose; LDL, low density lipoprotein; HDL, high density lipoprotein; GFR, glomerular filtration rate; TIBC, total iron binding capacity.
